# Editorial: Nutrition and diet practices: impact on body components and functioning

**DOI:** 10.3389/fendo.2023.1179751

**Published:** 2023-04-14

**Authors:** Roberta Zupo, Mikiko Watanabe, Giovanni De Pergola, Fabio Castellana

**Affiliations:** ^1^ Department of Interdisciplinary Medicine, University “Aldo Moro”, Bari, Italy; ^2^ Department of Experimental Medicine, Section of Medical Pathophysiology, Food Science and Endocrinology, Sapienza University of Rome, Rome, Italy; ^3^ Unit of Geriatrics and Internal Medicine, National Institute of Gastroenterology “Saverio de Bellis,” Research Hospital, Bari, Italy; ^4^ Unit of Data Sciences and Technology Innovation for Population Health, National Institute of Gastroenterology “Saverio de Bellis,” Research Hospital, Bari, Italy

**Keywords:** body composition, obesity, frailty, diet, sarcopenia

Nutritional status is a state of the body resulting from the balance of nutrient intake, absorption, and utilization and the influence of particular physiological and pathological states; it has strong repercussions in clinical practice and public health for being a key risk determinant of adverse health conditions.

To date, a large research window on the role of diet and nutritional state in characterizing body composition as a set of body components and related functional aspects remains open. This Research Topic has contributed to the understanding of the relationship between diet, nutritional status, and body composition features, in order to deepen the comprehension of the role of diet in influencing specific body composition and functioning in the context of well-defined disease states (physical frailty, sarcopenia, obesity, sarcopenic obesity, osteoporosis), or intermediate states.

The collection includes ten articles, mostly original research, and a single systematic review report. A graphical abstract of the research topic has been provided as **Figure 1**.

**Figure 1:**
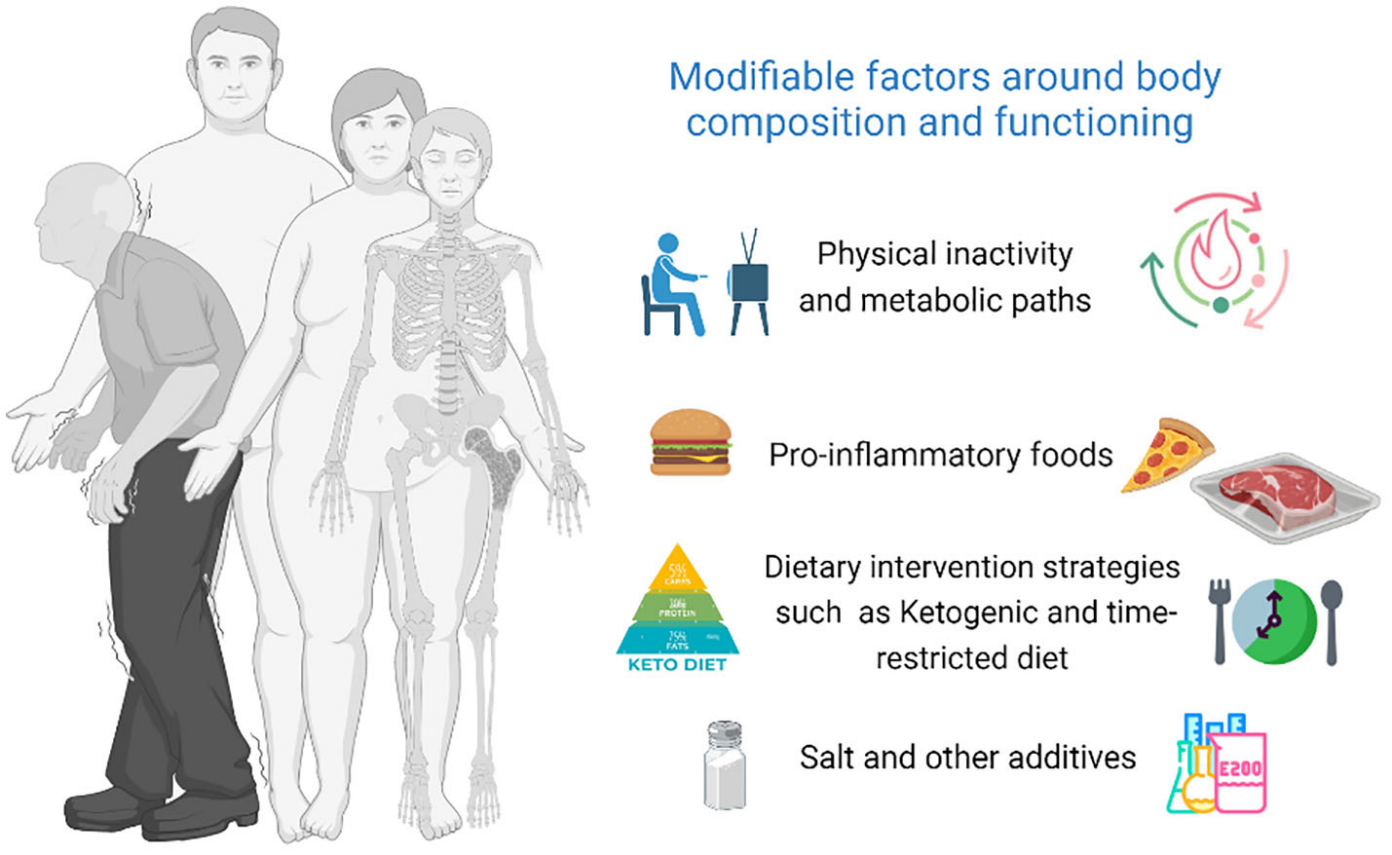
Graphical abstract of the Research Topic.

A large amount of the research focused on obesity as a target population phenotype. Among these, Zhang and Pu cross-sectionally investigated the level of body mass index (BMI) and waist circumference (WC) to gain the greatest bone mineral density (BMD) benefits in adults, using a representative sample over 50 from the NHANES, a major continuous cross-sectional survey in the United States. Of interest, they found a BMI saturation value, that is, for each unit rise in BMI over 24.3 kg/m2, the femoral neck BMD increases by 0.015 g/cm2. Interestingly, this is partially in contrast with previous literature, suggesting a U-shaped curve in the relationship between BMI and BMD (1). Again, overnutrition status was investigated in the study conducted by Hu et al. Here, some causal genetic relationship was investigated with genome-wide methodology (GWAS) between obesity and skin and soft tissue infections (SSTIs) using two-sample Mendelian randomization. Findings indicated a positive causal effect of obesity on an increased risk of SSTIs. This positive causality persisted after adjusting for the effects of type 2 diabetes (T2D) and peripheral vascular disease.

In the contexts of weight-loss strategies, studies by Ernesti et al. reported on predictors of weight loss upon a very-low-energy diet (VLED). A 45-day intervention of VLED, using a so called very-low calorie ketogenic diet (VLCKD) with meal replacement, was performed on a sample of 34 subjects, investigating those molecules involved in energy homeostasis and, more specifically, fibroblast growth factor 21 (FGF21) as an hepatokine of still unclear physiology. Findings indicated men with central obesity and a lower amount of circulating FGF21 to be key factors in reaping greater benefits in terms of weight loss achieved by following a VLCKD diet. Previous research showed that FGF21 is indeed crucial in determining the weight loss effect following a ketogenic diet in mouse models (2), although the physiology of FGF21 in human and murine models seems very different, suggesting that FGF21 is most likely not directly responsible for greater weight loss in human subjects. Moreover, although the effect on weight loss is widely recognized, to date no human studies with adequate and powerful experimental designs have been conducted to definitively understand the impact of ketogenic diet therapy on bone health, according to findings from another Italian research group (Garofalo et al.). Beyond VLCKD, a growing number of studies have explored intervention with time-restricted feeding (TRF). In this regard, Bao et al. conducted the first study to systematically quantify and compare energy balance during intervention with TRF in healthy subjects. Surprisingly, TRF was able to evoke significant fecal energy loss and urinary energy loss tendency without altering energy expenditure, causing a negative energy balance. These findings suggest TRF as an alternative dietary strategy for obesity, although it should be kept in mind that, according to current evidence, TRF seems equivalent to continuous energy restriction (3).

Moving on to other reports on dietary exposure with older target populations, sarcopenia, sarcopenic obesity, and frailty are well-known as important conditions highly prevalent in late life (4, 5). While frailty is a multisystem impairment associated with increased vulnerability to stressors, sarcopenia is the loss of muscle mass and function. As for frailty, this collection reported data on tea consumption in relation to physical frailty (Li et al.), indicating higher tea consumption being associated with lower prevalence of frailty in Chinese men aged 60-79 years, rural residents, and individuals participating in community activities (6).

As for sarcopenia, Chen et al. indicated that both depressive symptoms and the inflammatory potential of diet have direct and indirect effects on low muscle mass, grip strength, and muscle mass, through one and the other, in aging. This provides important insights for integrated nutritional and psychological strategies in the prevention of sarcopenia and loss of muscle in later life (7). This means, as already hypothesized in the literature, that sarcopenia is not only an age-related problem, but also associated with a number of long-term conditions even in early middle age. Comprehensive psychological and behavioral interventions, such as promoting an anti-inflammatory diet and improving mental health, have some potential to prevent sarcopenia early in life (8). Thus, screening is key to stratify risk, and nutritional tools should not be overlooked. the geriatric nutritional risk index has been used as a tool to assess nutrition status in the elderly, and Wang et al. found it to be positively correlated with femoral BMD and negatively correlated with osteoporosis risk in postmenopausal American women.

As for sarcopenic obesity, Kim et al. reported on single food item relationships to sarcopenic obesity outcomes. Here interest shifted to the condition of obesity accompanied by loss of muscle mass and functional impairment, that is, sarcopenic obesity. The target population were aging Koreans living in a rural area, who underwent a comprehensive nutritional and body composition assessment to assess how dietary factors, grip strength, body composition, and prevalence of sarcopenic obesity were associated with each other in a cross-sectional setting. Findings indicated excessive grain and meat consumption, as well as imbalanced macronutrient intake, to produce significant effects on the risk of prevalence of sarcopenic obesity by increasing the loss of muscle mass and/or body fat in older adults from Korea.

Last, and highly interesting, Liao et al.’s report indicated that in the population over 50, high dietary salt intake is associated with histone methylation in salt-sensitive individuals. This additional piece provides more evidence on how epigenetic mechanisms may influence gene expression and function while mediating crosstalk between genes and environment.

In conclusion, when discussing modifiable factors in contexts of noncommunicable diseases related to body composition and functioning such as obesity, sarcopenia, frailty, and osteoporosis, nutrition is key. Extensive room for research is left on the biological mechanisms underlying dietary strategies, individual food items, nutrition status, and dietary patterns able to shape physical outcome trajectories, especially in the growing aging population. Dietary inflammatory load, positive energy balance, and salt abuse are among the many habits to be monitored. As diet strategies to manage body composition, VLCKD ranks high on the list, but the recent research leaves room for further insights into innovative approaches such as time-restricted feeding.

## Author contributions

RZ and MW wrote the first draft of the manuscript; GP and FC read the manuscript and sent additional suggestions. All authors contributed to the article and approved the submitted version.
